# Supplementary figures and images for: Regulation of the plastochron by three many-noded dwarf genes in barley
Source: PLoS Genet. 2021 May 10;17(5):e1009292. doi: 10.1371/journal.pgen.1009292 (PMC8136844; doi:10.1371/journal.pgen.1009292)

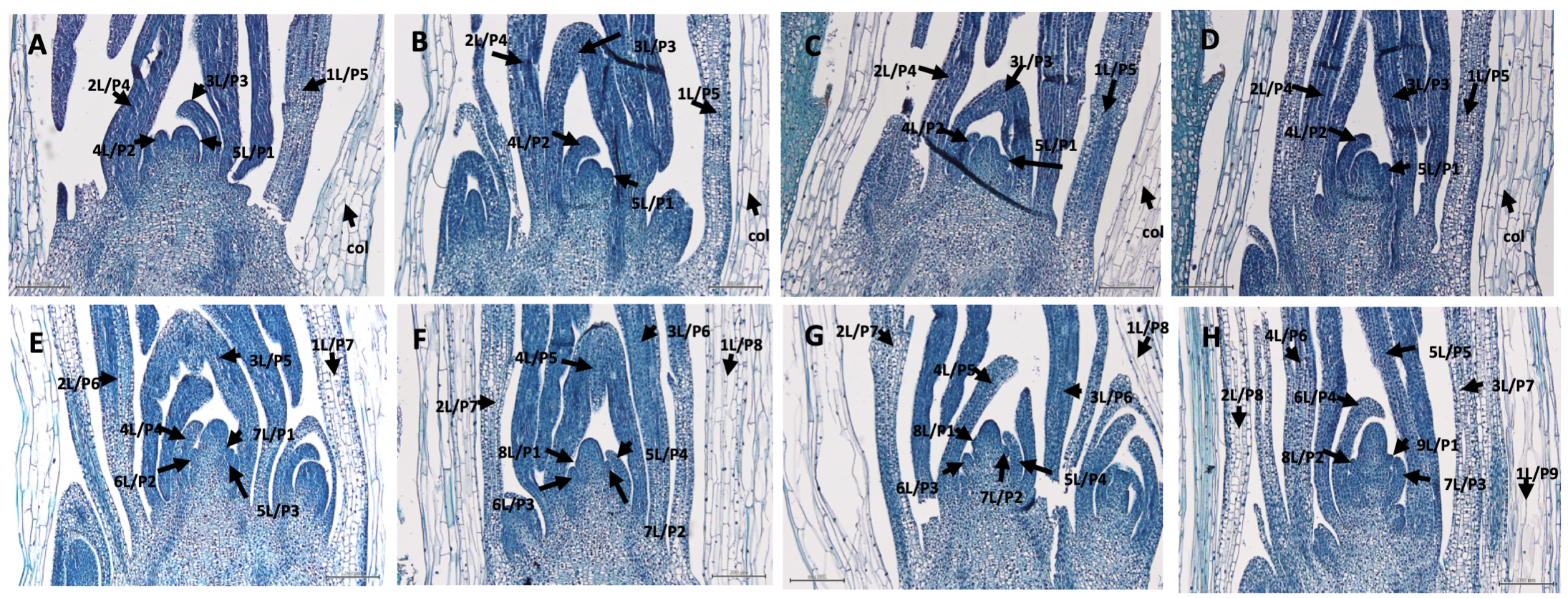

Supplement: S1 Fig — Inner structure of the wild-type and mnd mutants at 1 week (A–D) and 2 weeks (E–H) after germination. (A, E) Akashinriki, (B, F) mnd8OUM165, (C, G) mnd4OUM169, and (D, H) mnd1OUX051. xL indicates the xth leaf and Px indicates the order of leaf emergence from the shoot apical meristem. col, coleoptile. Bars: 200 μm. (TIF) [file pgen.1009292.s001.tif]

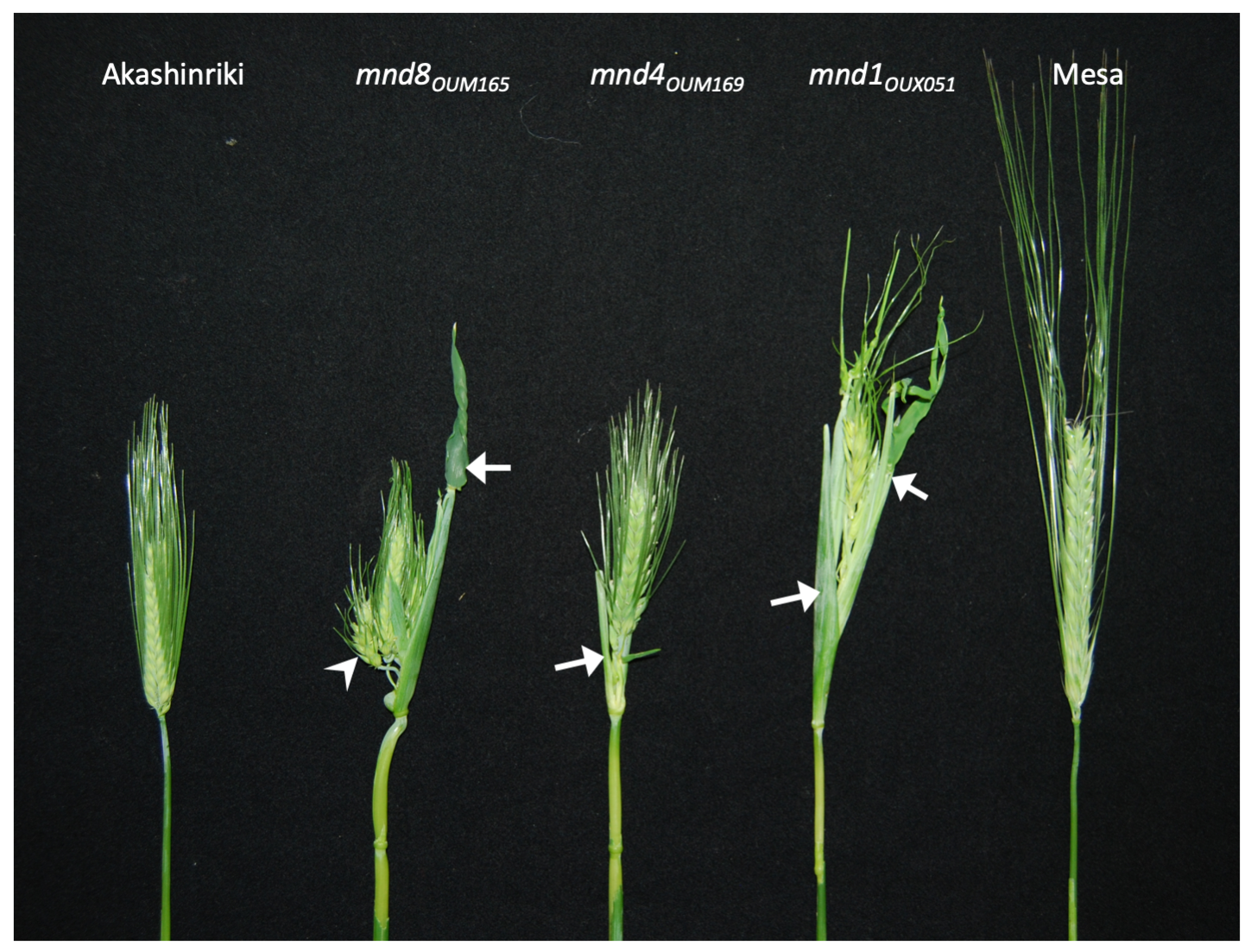

Supplement: S2 Fig — Arrows indicate elongated bracts or ectopic shoot-like structures; arrowheads indicate branched panicles. (TIF) [file pgen.1009292.s002.tif]

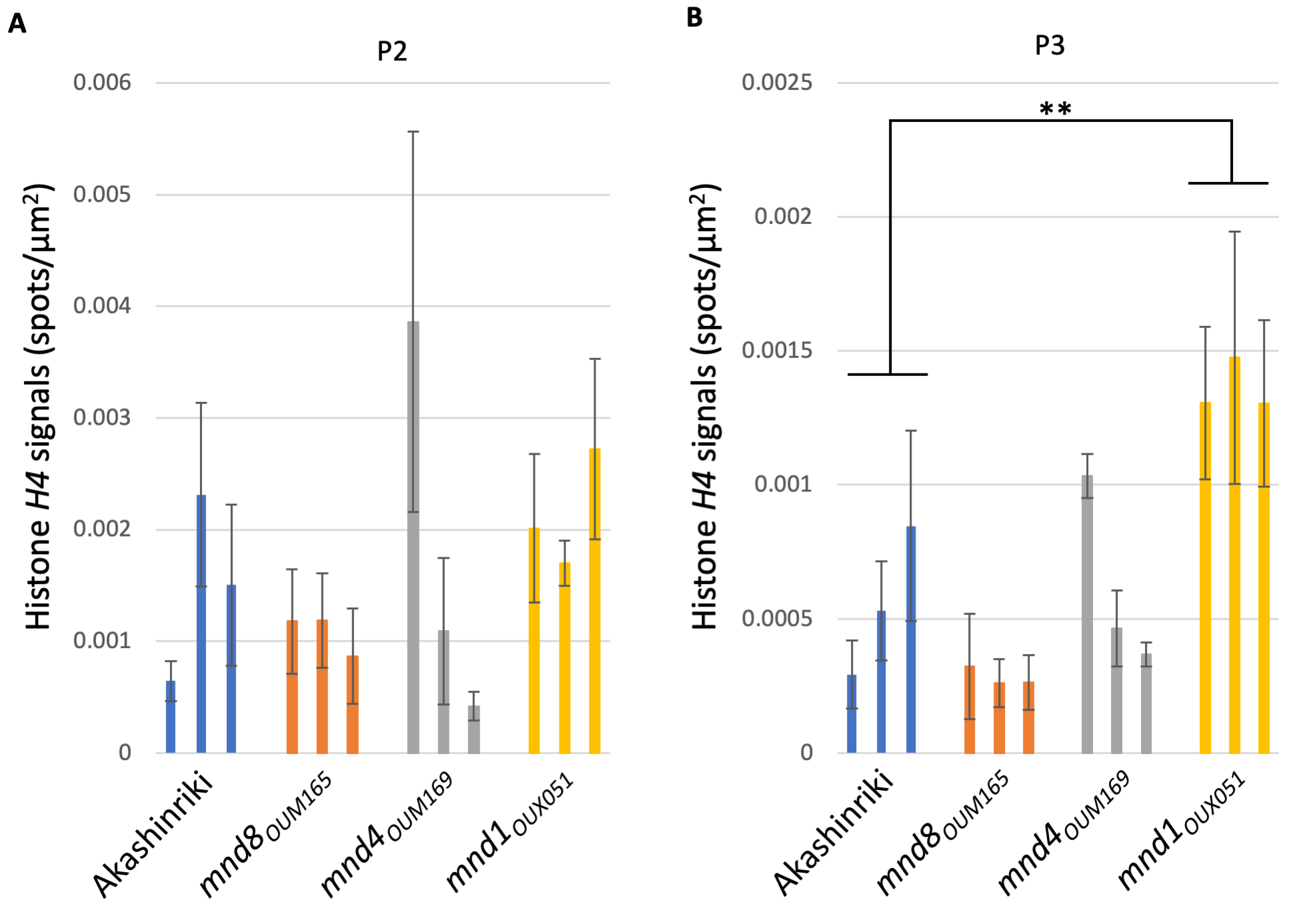

Supplement: S3 Fig — (A, B) The number of histone H4 spots per area of the leaf primordia were calculated using three independent samples of each genotype by in situ hybridization. (A) P2, (B) P3. Five serial sections in a sample were used for measurements. Data are shown as mean± SD. The area of the leaf primordium was measured with IMAGEJ (http://rsb.info.nih.gov/ij/).**P<0.01 (Student’s t-test). (TIF) [file pgen.1009292.s003.tif]

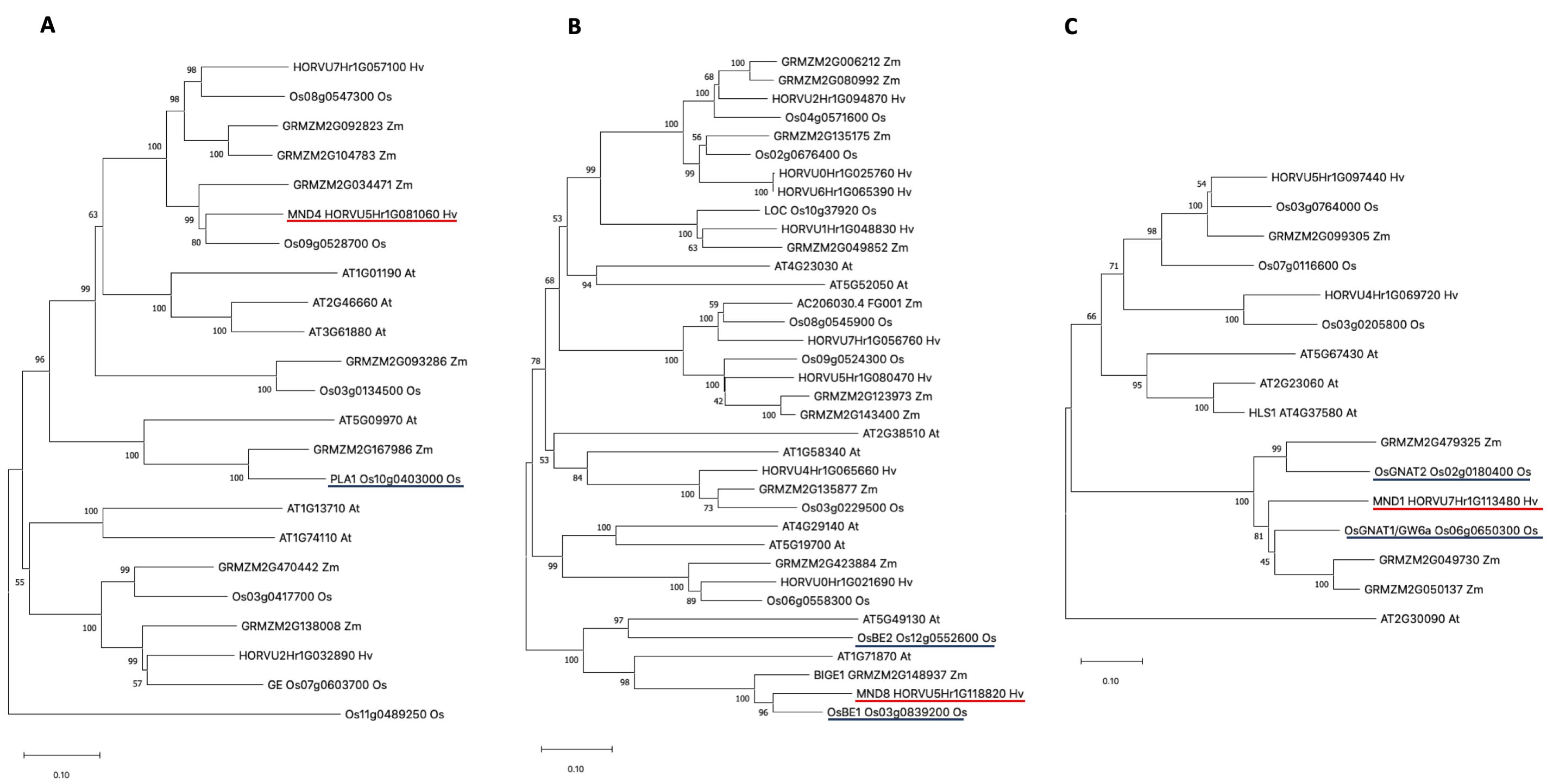

Supplement: S4 Fig — Phylogenetic tree of MND proteins from several angiosperms. (A) MND4, (B) MND8, and (C) MND1. Numbers above the branches are bootstrap values from 1,000 replicates. At, Arabidopsis thaliana; Zm, Zea mays; Os, Oryza sativa; Hv, Hordeum vulgare. Red and blue underlining indicates MND genes in barley and orthologs in rice, respectively. (TIF) [file pgen.1009292.s004.tif]

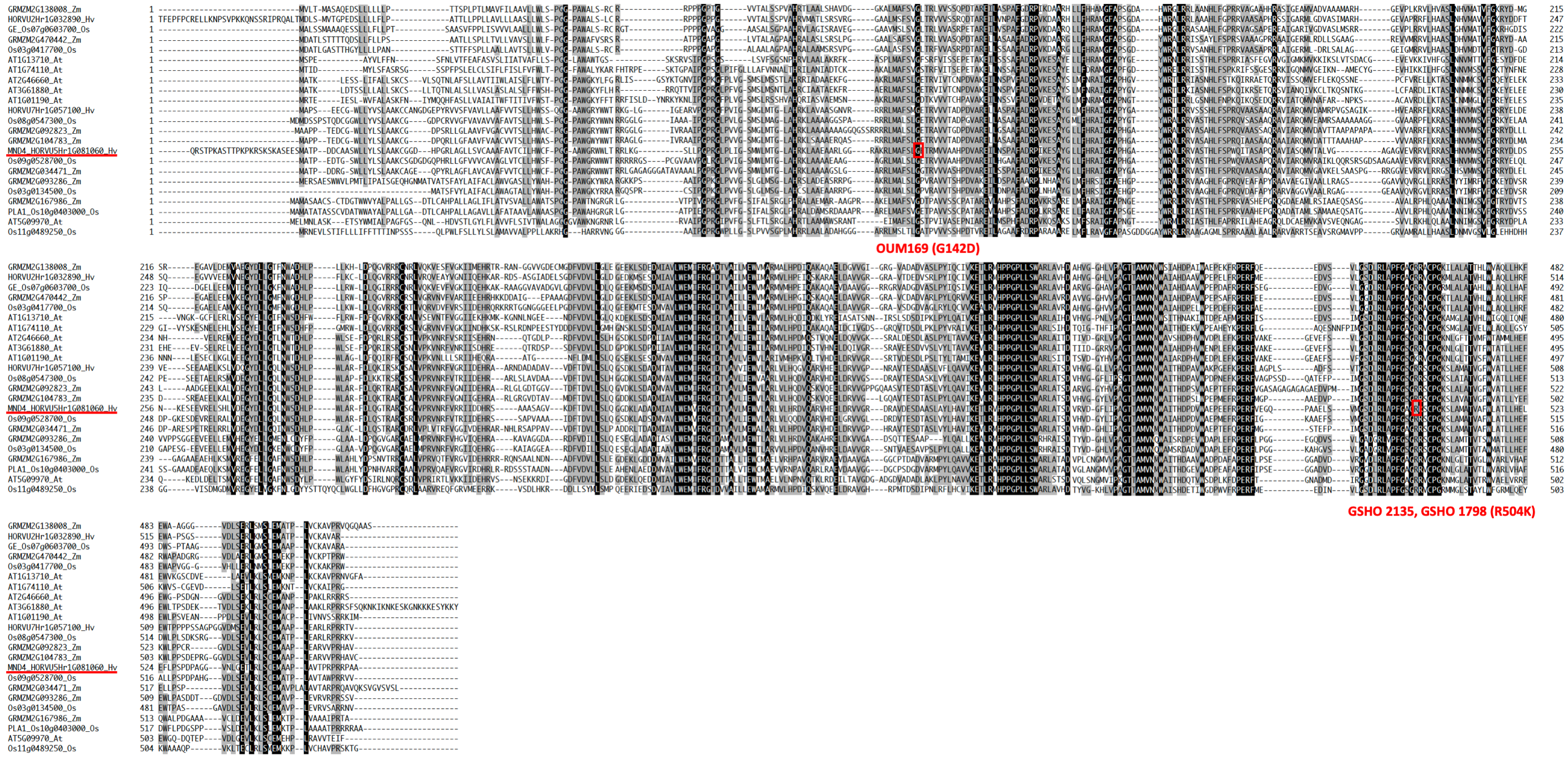

Supplement: S5 Fig — Alignment of MND4 and its homologous proteins in several angiosperms used in S4 Fig. The effect of each mnd mutation is indicated in red. Black and gray, 100% and more than 50% identical amino acids, respectively. At, Arabidopsis thaliana; Zm, Zea mays; Os, Oryza sativa; Hv, Hordeum vulgare (TIF) [file pgen.1009292.s005.tif]

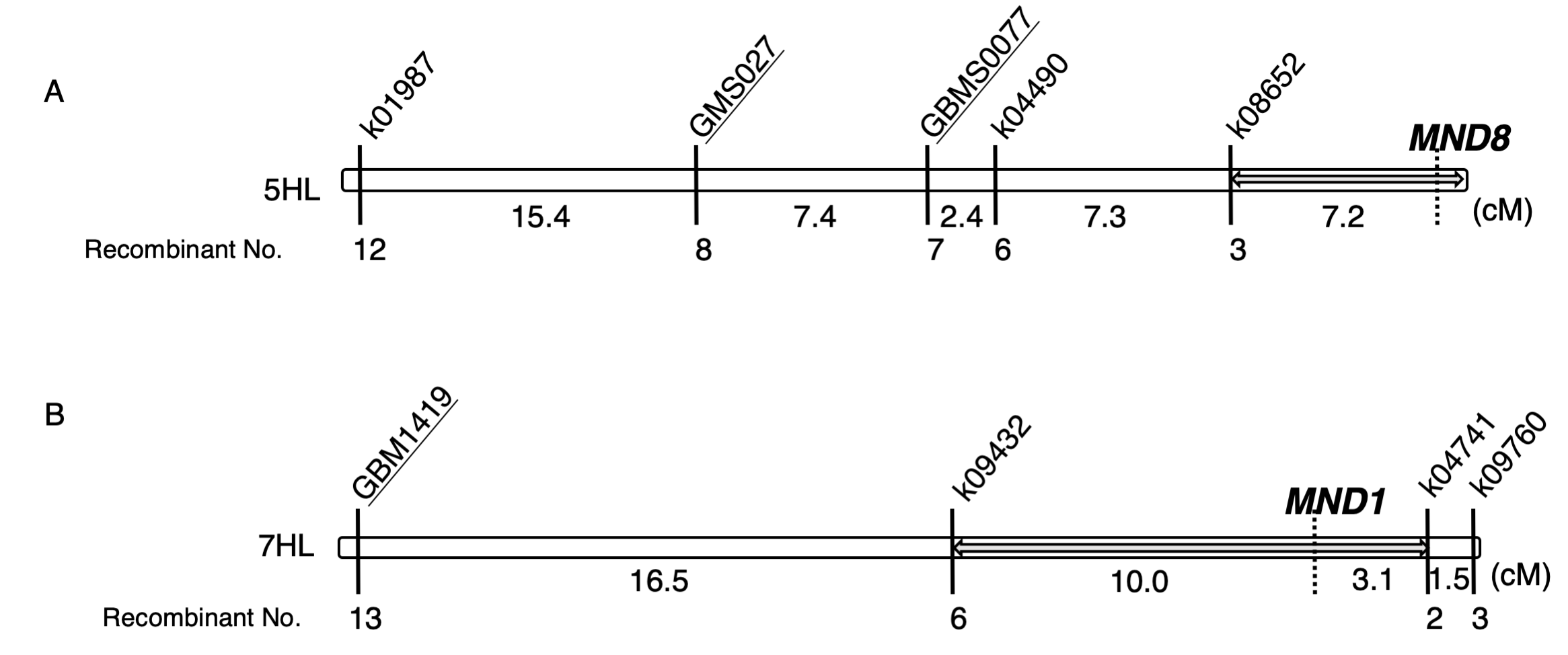

Supplement: S6 Fig — (A) For genetic mapping of MND8, 21 recessive homozygous F2 plants from a cross between Haruna Nijo and OUM165 were used. (B) For genetic mapping of MND1, 33 recessive homozygous F2 plants from a cross between OUI026 and SM6 were used. The double-ended arrows indicate the gene regions found in the mapping. EST markers with initial letter k were those reported by Sato et al. (2009). SSR markers underlined were developed by Varshney et al. (2007). MapMaker version 2.0 was used for map construction with LOD score 3. (TIF) [file pgen.1009292.s006.tif]

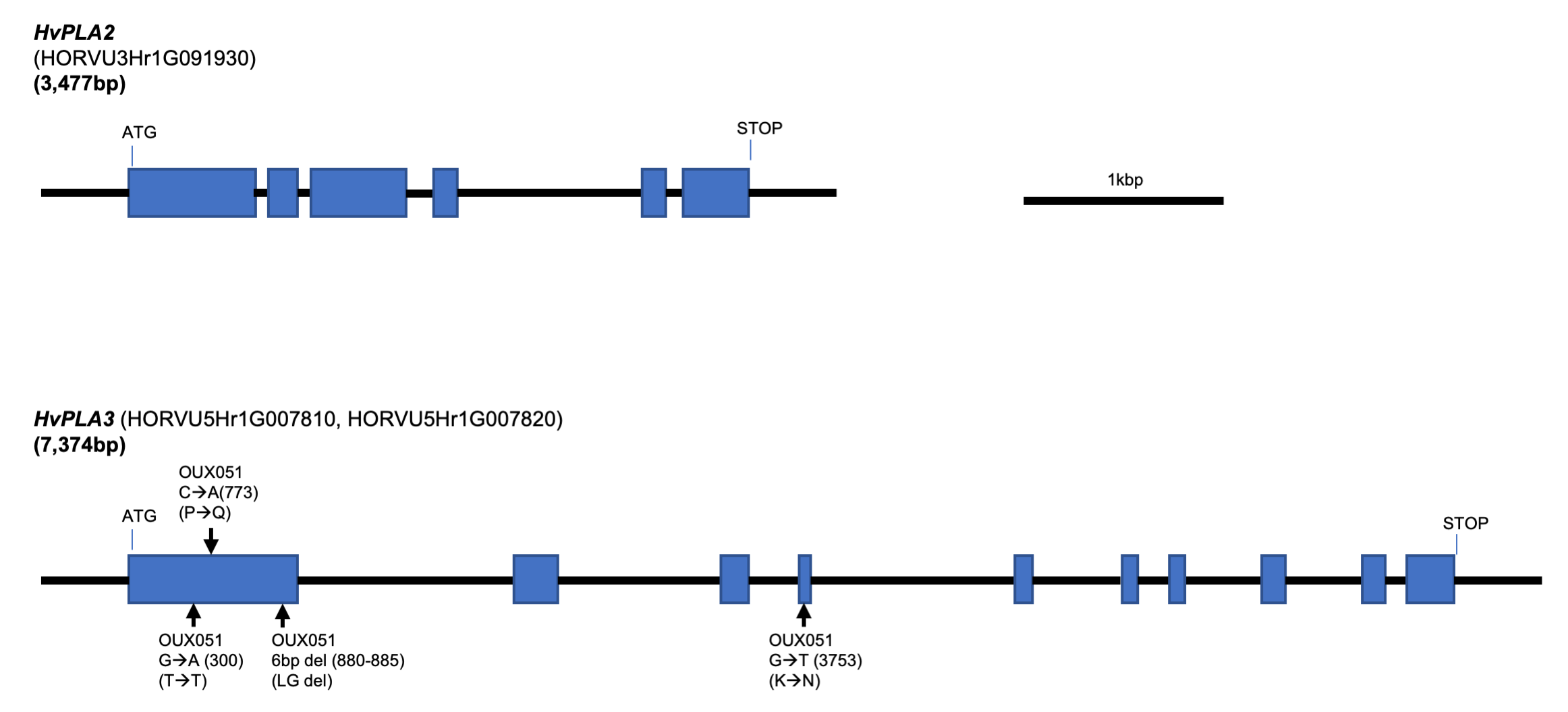

Supplement: S7 Fig — Boxes indicate exons. DNA polymorphisms between Akashinriki and OUX051 in the HvPLA3 genomic structure are indicated by arrows. (TIF) [file pgen.1009292.s007.tif]

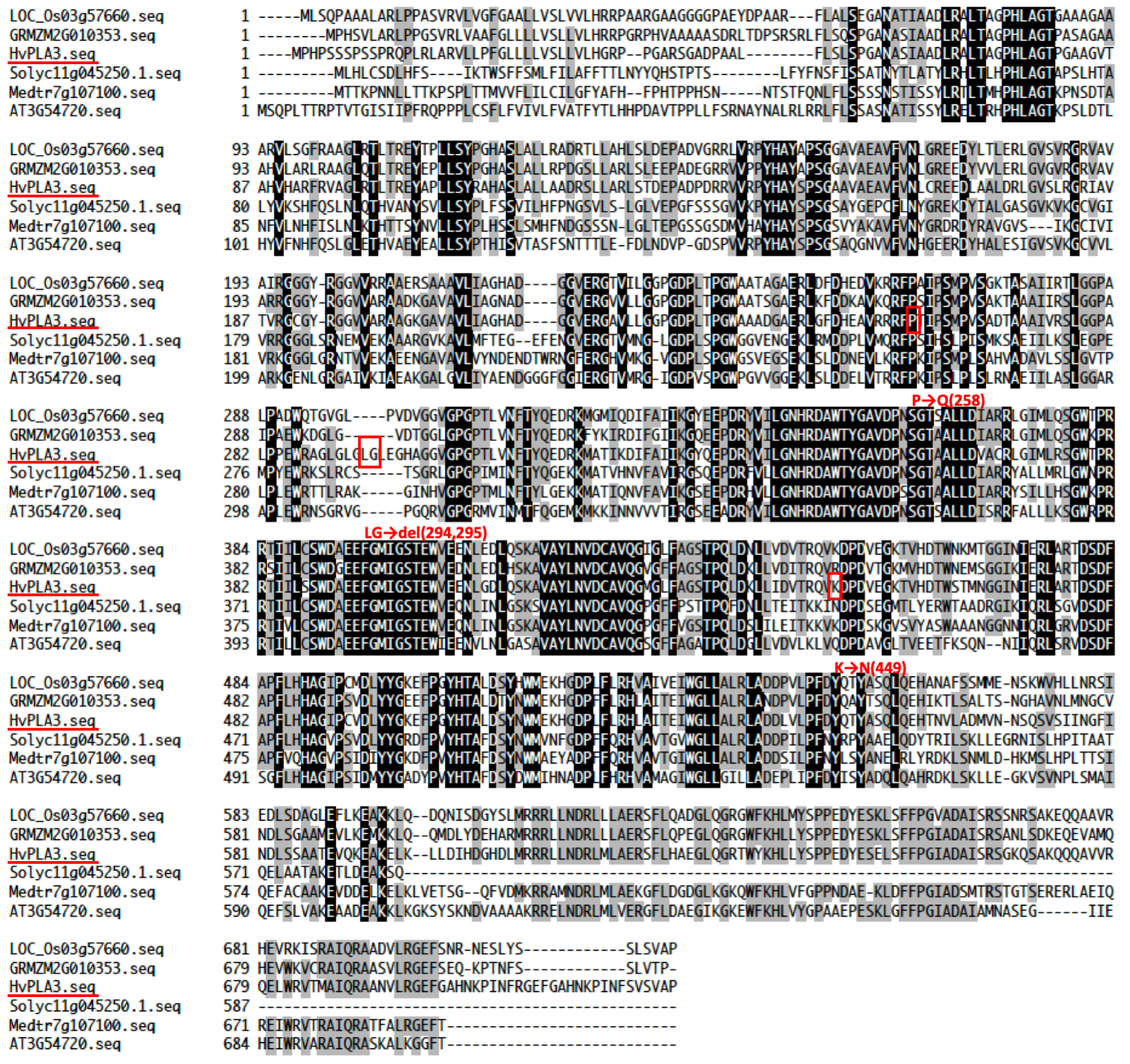

Supplement: S8 Fig — Alignment of HvPLA3 and its homologs in several angiosperms. The effects of mutations in OUX051 are indicated in red. Black and gray, 100% and more than 50% identical amino acids, respectively. LOC, Oryza sativa; GRMZM, Zea mays; Solyc, Solanum lycopersicum; Medtr, Medicago truncatula; AT, Arabidopsis thaliana. (TIF) [file pgen.1009292.s008.tif]

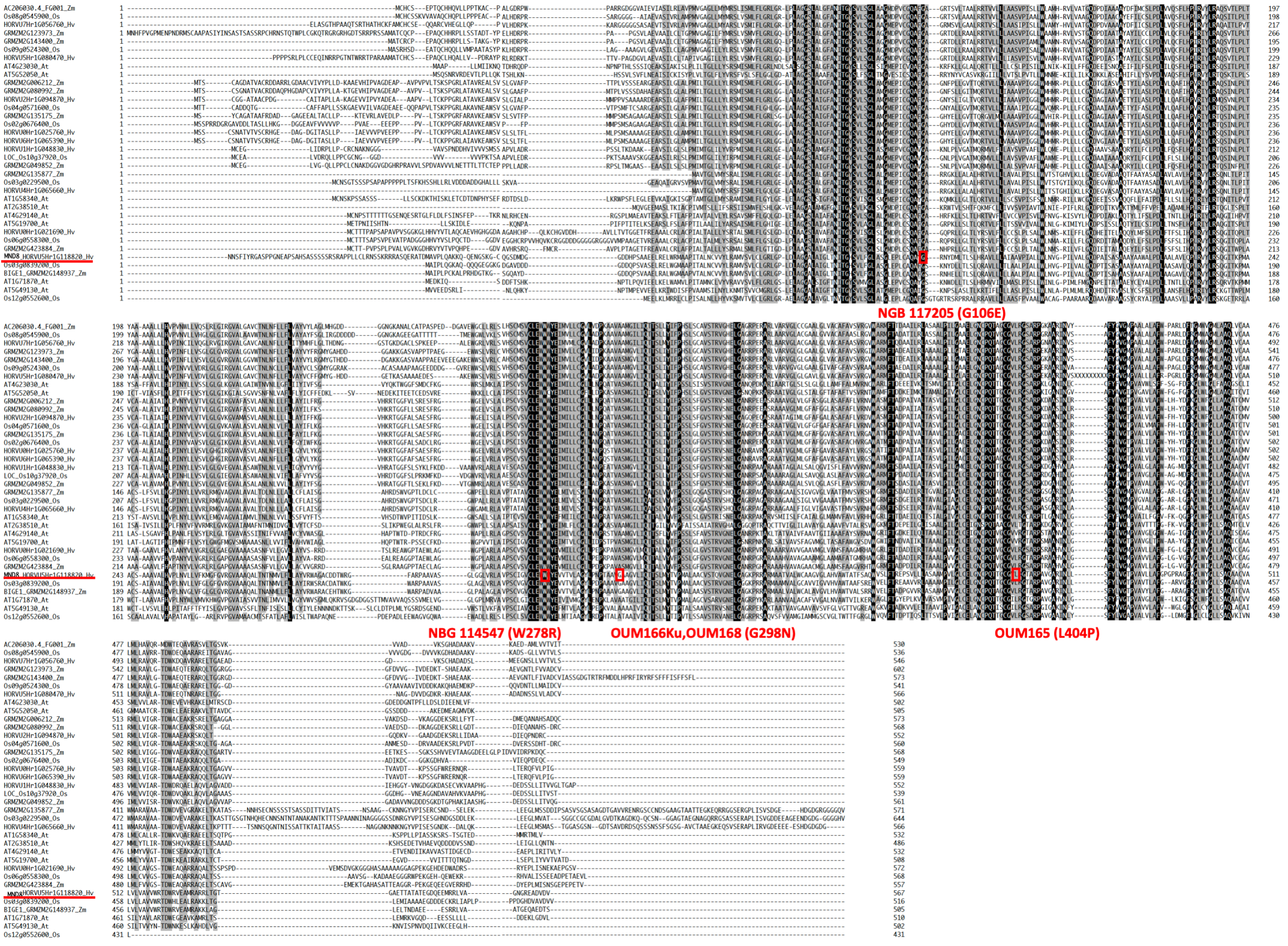

Supplement: S9 Fig — Alignment of MND8 and its homologous proteins in several angiosperms used in S4 Fig. The effect of each mnd mutation is indicated in red. Black and gray, 100% and more than 50% identical amino acids, respectively. At, Arabidopsis thaliana; Zm, Zea mays; Os, Oryza sativa; Hv, Hordeum vulgare. (TIF) [file pgen.1009292.s009.tif]

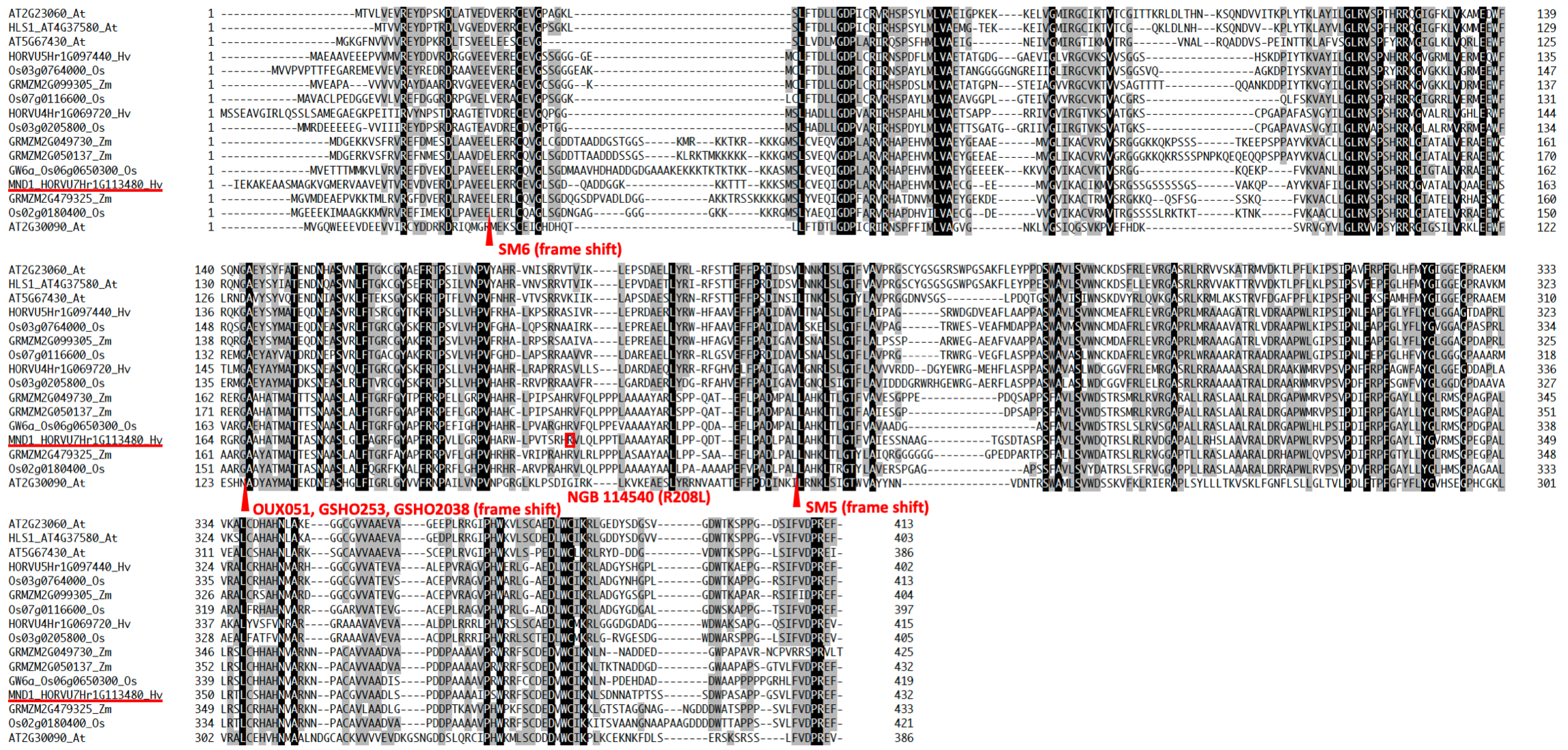

Supplement: S10 Fig — Alignment of MND1 and its homologs in the angiosperms used in S4 Fig. The effect of each mnd mutation is indicated in red. Black and gray, 100% and more than 50% identical amino acids, respectively. At, Arabidopsis thaliana; Zm, Zea mays; Os, Oryza sativa; Hv, Hordeum vulgare. (TIF) [file pgen.1009292.s010.tif]

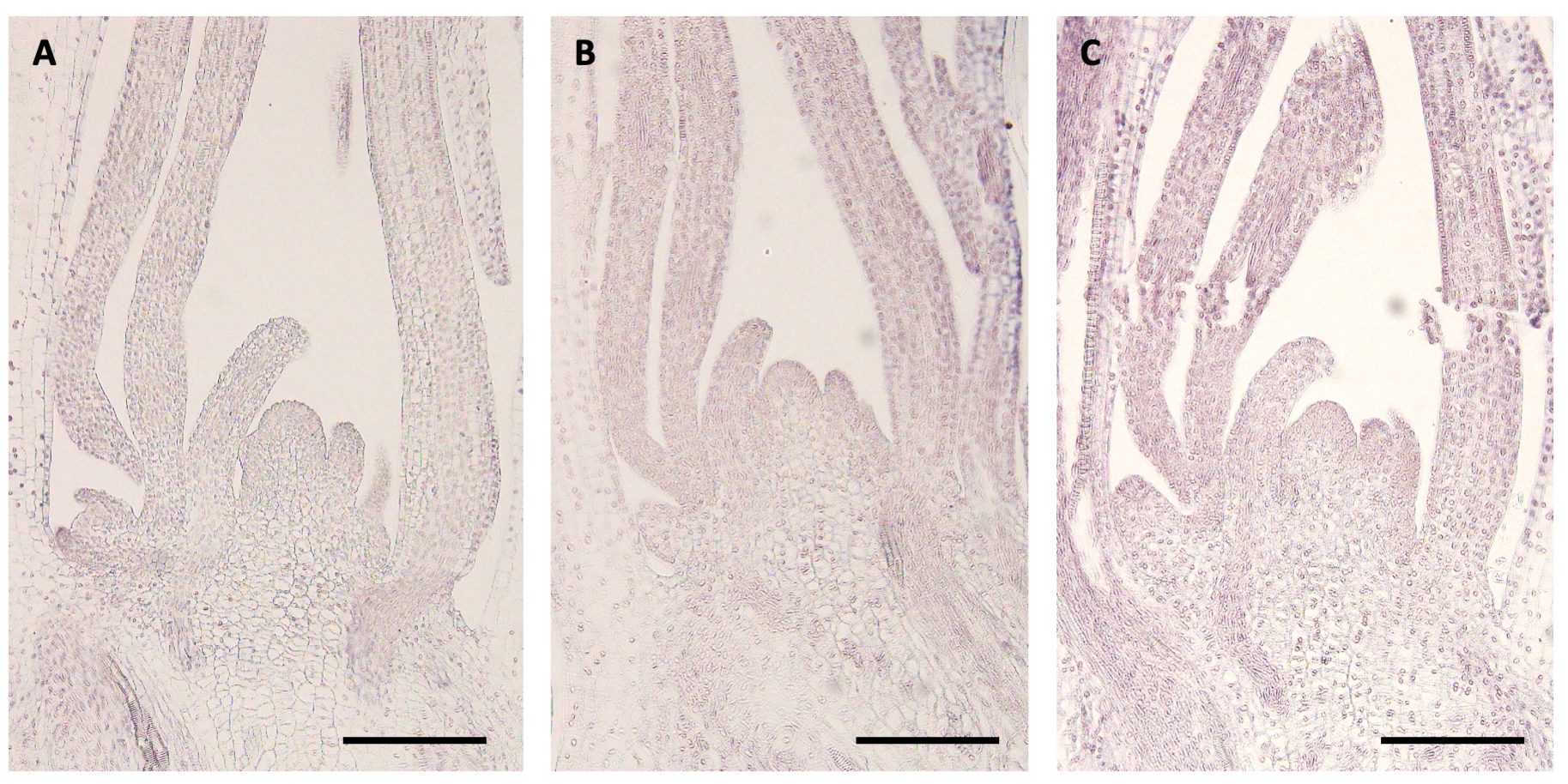

Supplement: S11 Fig — Shoot samples at 10 days after germination were used. (A) MND4, (B) MND8, and (C) MND1. Bars: 200 μm. (TIF) [file pgen.1009292.s011.tif]

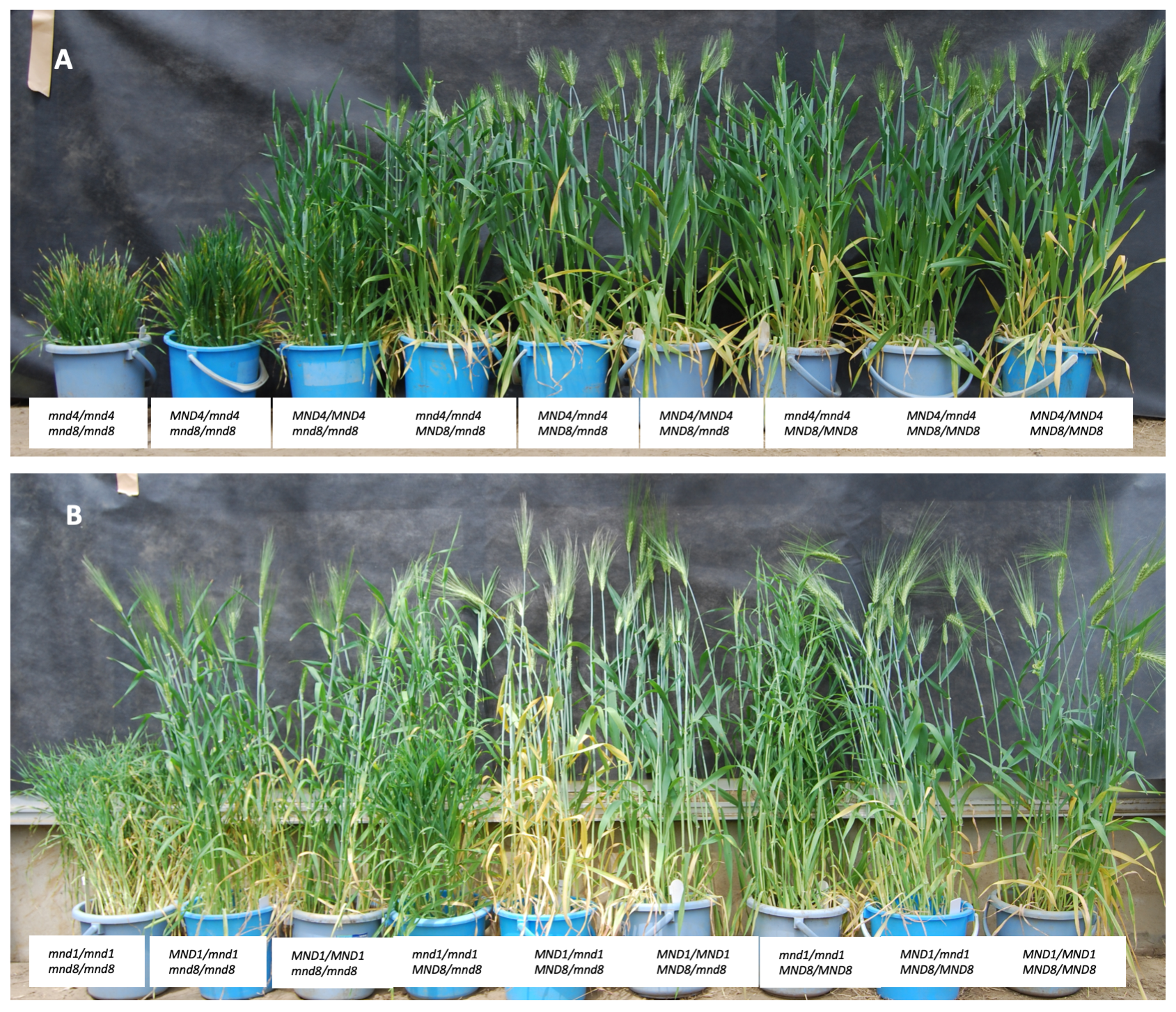

Supplement: S12 Fig — (A) Segregated plants of the F3 population derived from the F2 seeds of mnd4 OUM169 × mnd8OUM165 crossings. (B) Segregated plants of the F3 population derived from the F2 seeds of mnd1OUX051 × mnd8OUM165 crossings. The genotypes of the plants are indicated. (TIF) [file pgen.1009292.s012.tif]

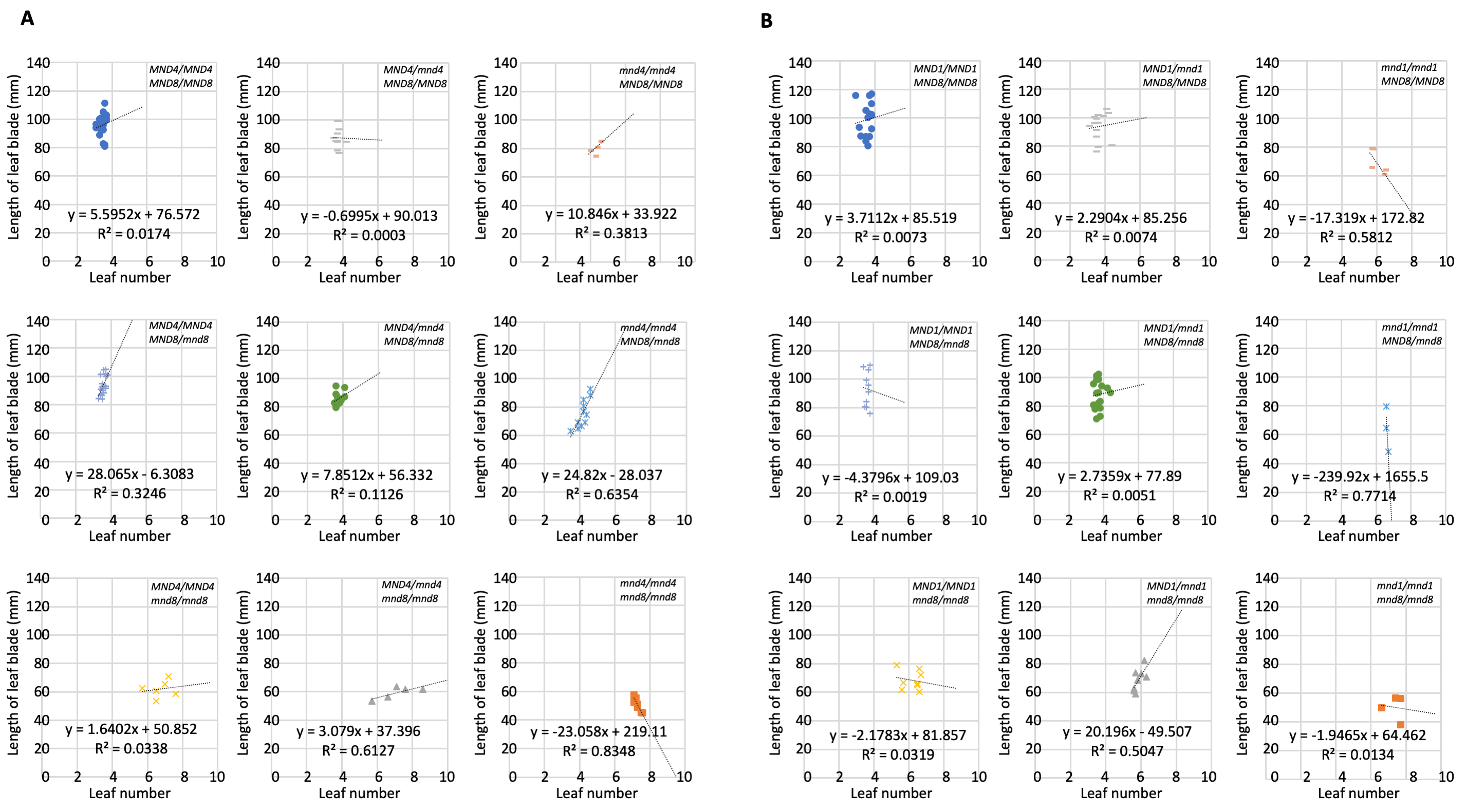

Supplement: S13 Fig — (A, B) Scatter plots of leaf number at 2 months after germination and the length of the second leaf blade with the same genotype from mnd4 OUM169 × mnd8OUM165 (A), and mnd1OUX051 × mnd8OUM165 (B) crossings. The linear regression line and the coefficient of determination (R2) are indicated. (TIF) [file pgen.1009292.s013.tif]

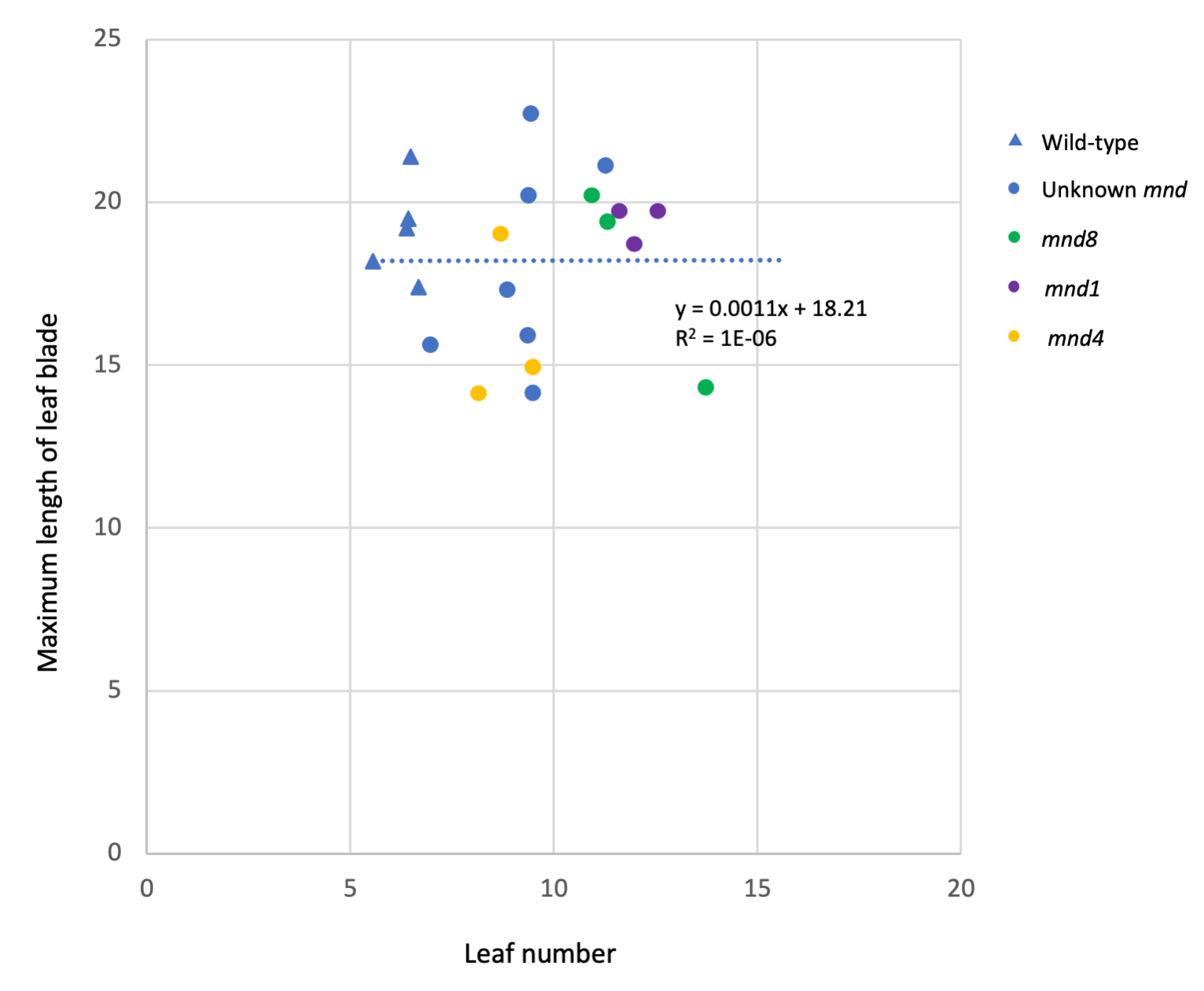

Supplement: S14 Fig — Scatter plot of leaf number at 100 days after germination and the maximum length of the leaf blade. Average values of the traits of five plants were used. The linear regression line and coefficient of determination (R2) are indicated. (TIF) [file pgen.1009292.s014.tif]

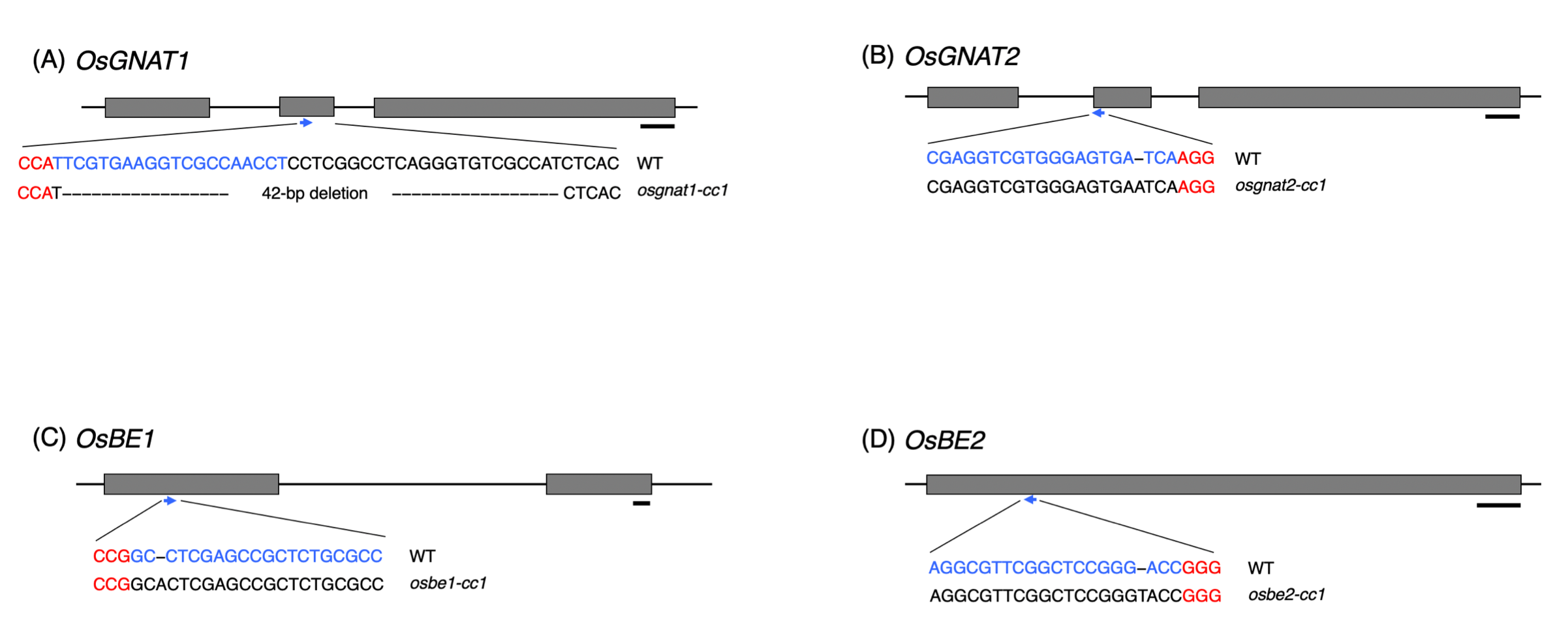

Supplement: S15 Fig — (A–D) Genomic structures and target sites of rice MND orthologs. (A) OsGNAT1, (B) OsGNAT2, (C) OsBE1, and (D) OsBE2. Boxes indicate exons. Arrows indicate target sites (protospacer adjacent motif [PAM] sequences [red characters] and guide sequences [blue characters]) and directions starting from each PAM sequence. The lower sequence is that of the mutant used in the experiment. Bars: 500 bp. (TIF) [file pgen.1009292.s015.tif]

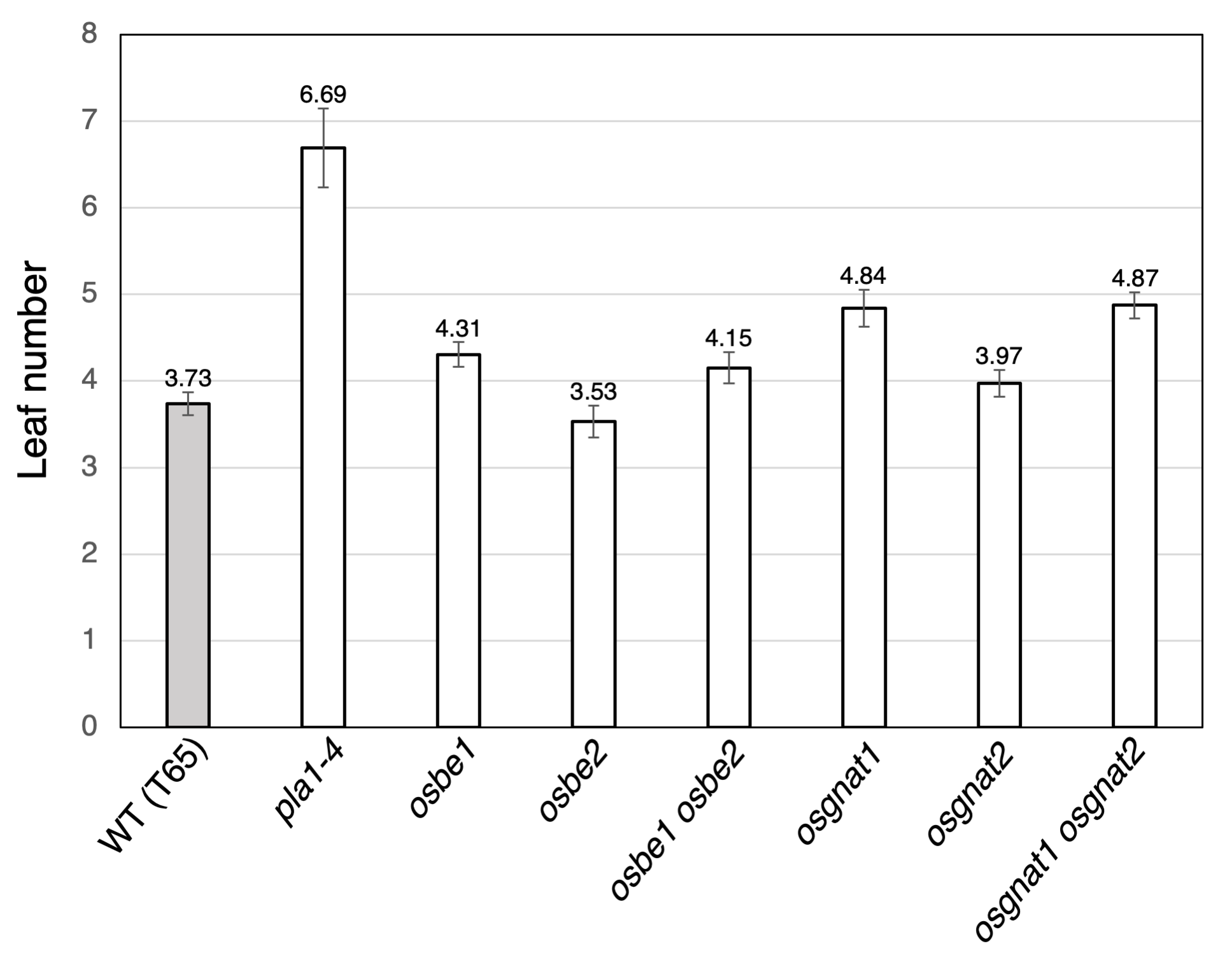

Supplement: S16 Fig — Data are presented as the means ± SDs (n ≥ 5). (TIF) [file pgen.1009292.s016.tif]

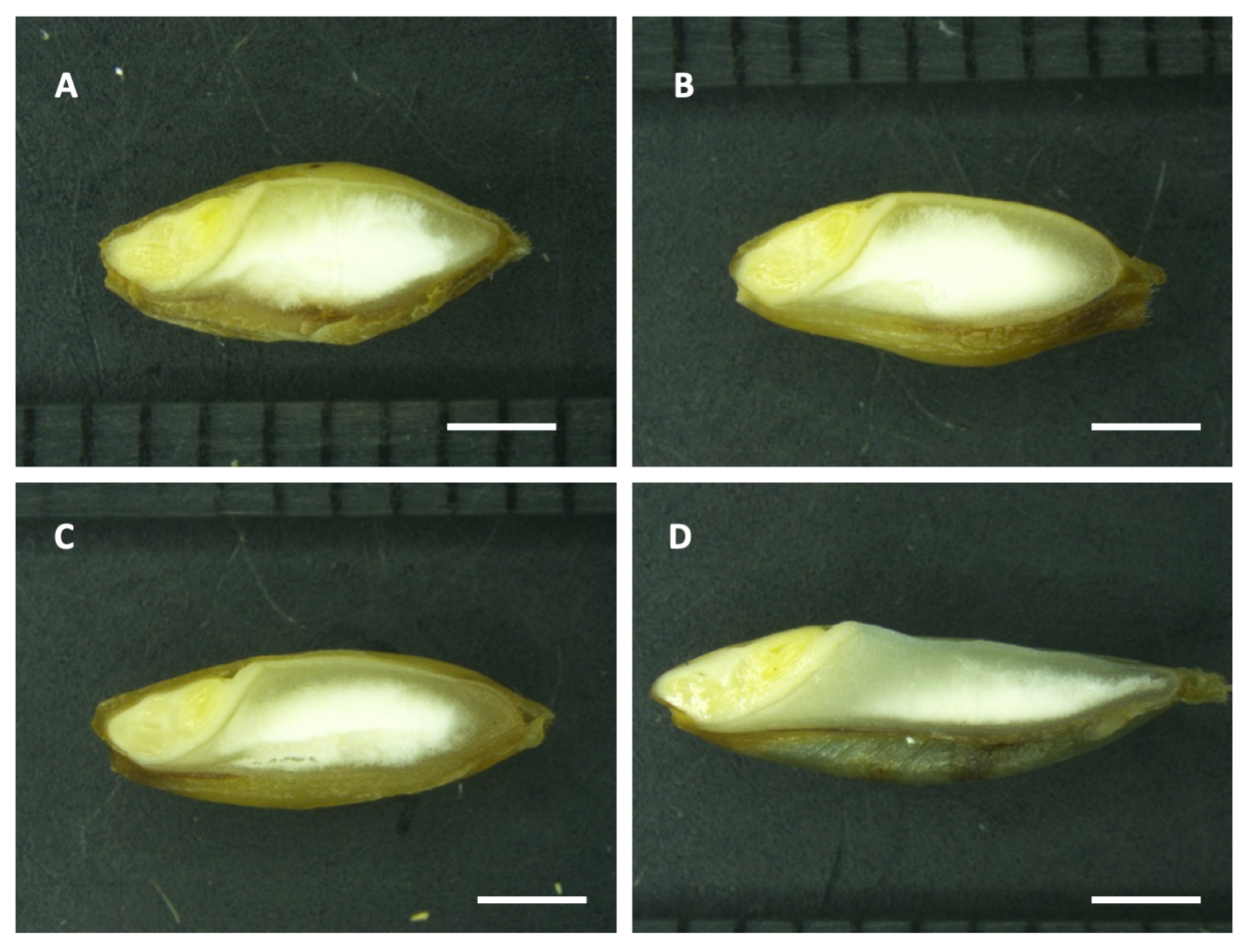

Supplement: S17 Fig — (A) Akashinriki, (B) mnd4 OUM169, (C) mnd8OUM165, and (D) mnd1OUX051. Bars: 2 mm. (TIF) [file pgen.1009292.s017.tif]
