# Supplementary material for: Regulation of the plastochron by three many-noded dwarf genes in barley
Source: PLoS Genet. 2021 May 10;17(5):e1009292. doi: 10.1371/journal.pgen.1009292 (PMC8136844; doi:10.1371/journal.pgen.1009292)
Supplement: S2 Table — (PPTX) [file pgen.1009292.s019.pptx]

## Slide 1
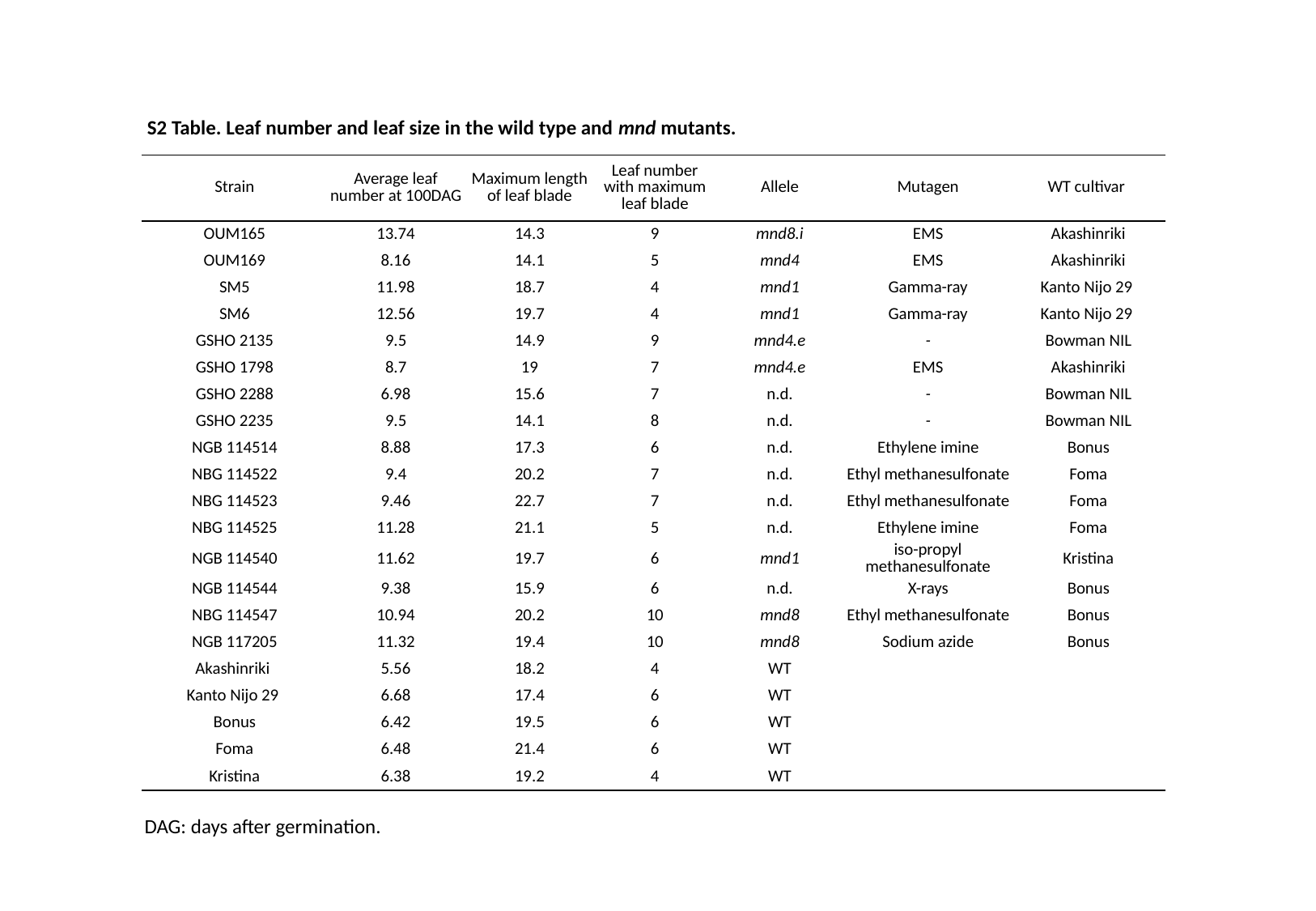

S2 Table. Leaf number and leaf size in the wild type and mnd mutants.
| Strain | Average leaf number at 100DAG | Maximum length of leaf blade | Leaf number with maximum leaf blade | Allele | Mutagen | WT cultivar |
| --- | --- | --- | --- | --- | --- | --- |
| OUM165 | 13.74 | 14.3 | 9 | mnd8.i | EMS | Akashinriki |
| OUM169 | 8.16 | 14.1 | 5 | mnd4 | EMS | Akashinriki |
| SM5 | 11.98 | 18.7 | 4 | mnd1 | Gamma-ray | Kanto Nijo 29 |
| SM6 | 12.56 | 19.7 | 4 | mnd1 | Gamma-ray | Kanto Nijo 29 |
| GSHO 2135 | 9.5 | 14.9 | 9 | mnd4.e | - | Bowman NIL |
| GSHO 1798 | 8.7 | 19 | 7 | mnd4.e | EMS | Akashinriki |
| GSHO 2288 | 6.98 | 15.6 | 7 | n.d. | - | Bowman NIL |
| GSHO 2235 | 9.5 | 14.1 | 8 | n.d. | - | Bowman NIL |
| NGB 114514 | 8.88 | 17.3 | 6 | n.d. | Ethylene imine | Bonus |
| NBG 114522 | 9.4 | 20.2 | 7 | n.d. | Ethyl methanesulfonate | Foma |
| NBG 114523 | 9.46 | 22.7 | 7 | n.d. | Ethyl methanesulfonate | Foma |
| NBG 114525 | 11.28 | 21.1 | 5 | n.d. | Ethylene imine | Foma |
| NGB 114540 | 11.62 | 19.7 | 6 | mnd1 | iso-propyl methanesulfonate | Kristina |
| NGB 114544 | 9.38 | 15.9 | 6 | n.d. | X-rays | Bonus |
| NBG 114547 | 10.94 | 20.2 | 10 | mnd8 | Ethyl methanesulfonate | Bonus |
| NGB 117205 | 11.32 | 19.4 | 10 | mnd8 | Sodium azide | Bonus |
| Akashinriki | 5.56 | 18.2 | 4 | WT | | |
| Kanto Nijo 29 | 6.68 | 17.4 | 6 | WT | | |
| Bonus | 6.42 | 19.5 | 6 | WT | | |
| Foma | 6.48 | 21.4 | 6 | WT | | |
| Kristina | 6.38 | 19.2 | 4 | WT | | |
DAG: days after germination.
