# Supplementary material for: Regulation of the plastochron by three many-noded dwarf genes in barley
Source: PLoS Genet. 2021 May 10;17(5):e1009292. doi: 10.1371/journal.pgen.1009292 (PMC8136844; doi:10.1371/journal.pgen.1009292)
Supplement: S3 Table — (PPTX) [file pgen.1009292.s020.pptx]

## Slide 1
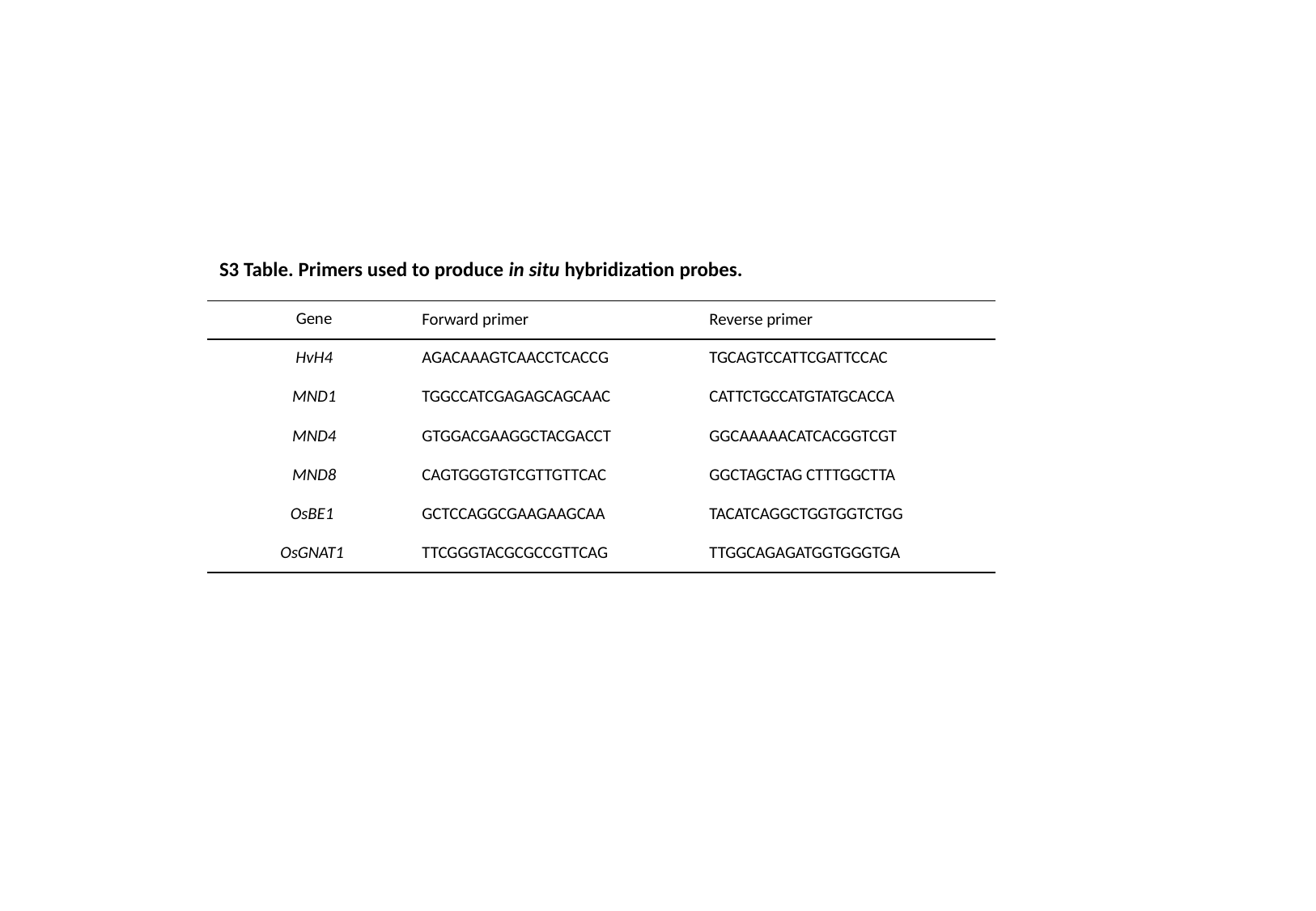

S3 Table. Primers used to produce in situ hybridization probes.
| Gene | Forward primer | Reverse primer |
| --- | --- | --- |
| HvH4 | AGACAAAGTCAACCTCACCG | TGCAGTCCATTCGATTCCAC |
| MND1 | TGGCCATCGAGAGCAGCAAC | CATTCTGCCATGTATGCACCA |
| MND4 | GTGGACGAAGGCTACGACCT | GGCAAAAACATCACGGTCGT |
| MND8 | CAGTGGGTGTCGTTGTTCAC | GGCTAGCTAG CTTTGGCTTA |
| OsBE1 | GCTCCAGGCGAAGAAGCAA | TACATCAGGCTGGTGGTCTGG |
| OsGNAT1 | TTCGGGTACGCGCCGTTCAG | TTGGCAGAGATGGTGGGTGA |
