# Supplementary material for: Regulation of the plastochron by three many-noded dwarf genes in barley
Source: PLoS Genet. 2021 May 10;17(5):e1009292. doi: 10.1371/journal.pgen.1009292 (PMC8136844; doi:10.1371/journal.pgen.1009292)
Supplement: S4 Table — (PPTX) [file pgen.1009292.s021.pptx]

## Slide 1
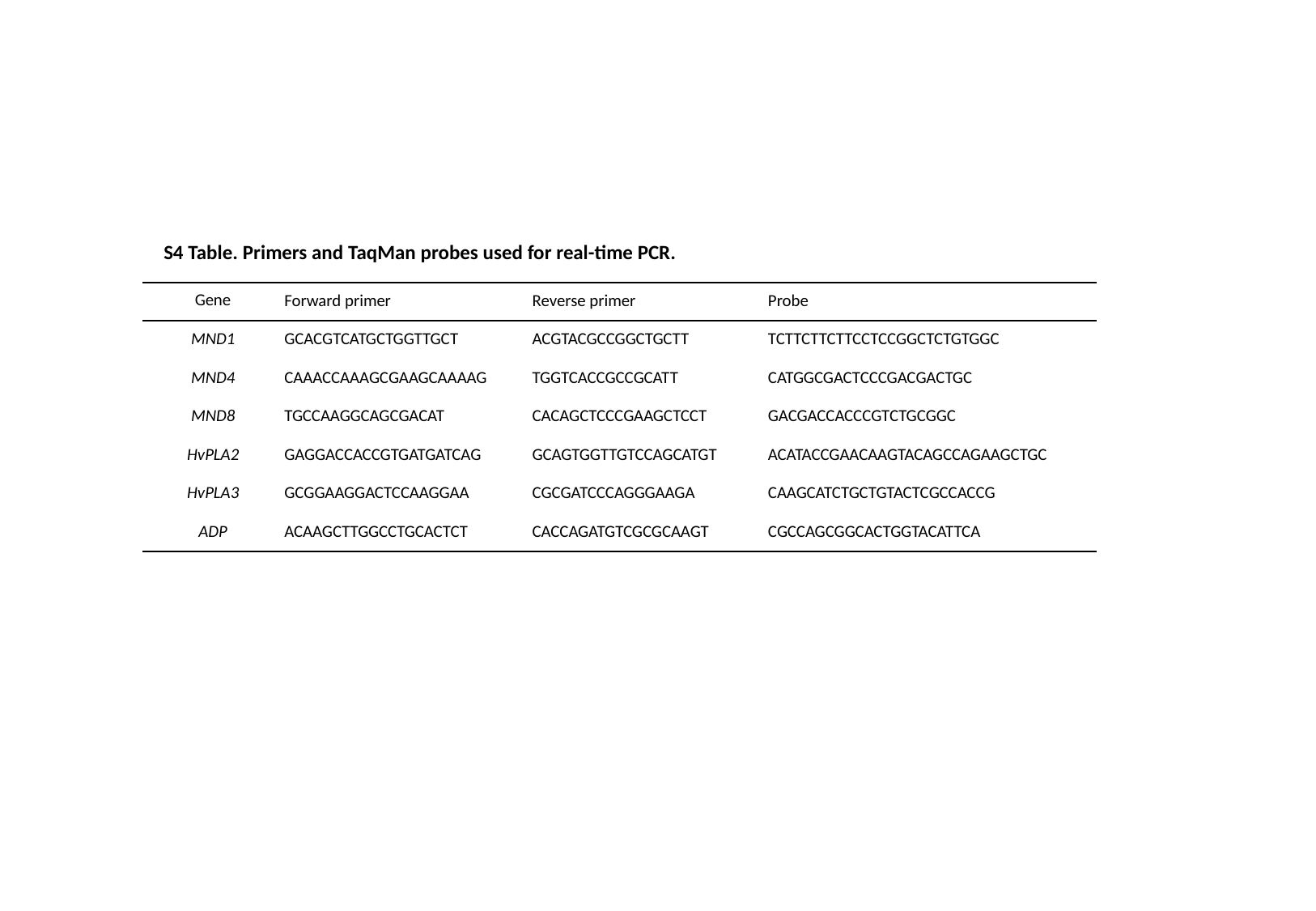

S4 Table. Primers and TaqMan probes used for real-time PCR.
| Gene | Forward primer | Reverse primer | Probe |
| --- | --- | --- | --- |
| MND1 | GCACGTCATGCTGGTTGCT | ACGTACGCCGGCTGCTT | TCTTCTTCTTCCTCCGGCTCTGTGGC |
| MND4 | CAAACCAAAGCGAAGCAAAAG | TGGTCACCGCCGCATT | CATGGCGACTCCCGACGACTGC |
| MND8 | TGCCAAGGCAGCGACAT | CACAGCTCCCGAAGCTCCT | GACGACCACCCGTCTGCGGC |
| HvPLA2 | GAGGACCACCGTGATGATCAG | GCAGTGGTTGTCCAGCATGT | ACATACCGAACAAGTACAGCCAGAAGCTGC |
| HvPLA3 | GCGGAAGGACTCCAAGGAA | CGCGATCCCAGGGAAGA | CAAGCATCTGCTGTACTCGCCACCG |
| ADP | ACAAGCTTGGCCTGCACTCT | CACCAGATGTCGCGCAAGT | CGCCAGCGGCACTGGTACATTCA |
